# Renal Effects of High-Dose Versus Low-Dose Lisinopril in Patients With Diabetic Nephropathy

**DOI:** 10.7759/cureus.29873

**Published:** 2022-10-03

**Authors:** Farah R Rashid, Muhammad Abubakar, Hafsa Fayyaz, Naseem Umer, Anum Shafiq, Waseem Sajjad, Khalifa Rashid, Aayat Ellahi

**Affiliations:** 1 Department of Medicine, Khyber Teaching Hospital, Peshawar, PAK; 2 Department of Medicine, Holy Family Hospital, Rawalpindi, PAK; 3 Department of Medicine, Sir Ganga Ram Hospital, Lahore, PAK; 4 Department of Medicine, Hamad General Hospital, Doha, QAT; 5 Department of Medicine, Lady Reading Hospital, Peshawar, PAK; 6 Department of Medicine, Ayub Teaching Hospital, Abbotabad, PAK; 7 Department of Emergency Medicine, Hayatabad Medical Complex (HMC), Peshawar, PAK; 8 Department of Medicine, Rehman Medical College, Peshawar, PAK; 9 Department of Community Medicine, Jinnah Sindh Medical University, Karachi, PAK

**Keywords:** microalbuminuria, lisinopril, cardiovascular disease, angiotensin converting enzyme inhibitor, arb, acei

## Abstract

Background

The present study was conducted to assess the renal effects of high dose versus low dose lisinopril in patients with diabetic nephropathy.

Methodology

A prospective observational study was conducted at the Khyber Teaching Hospital, Peshawar, Khyber Pakhtunkhwa, Pakistan, between July 1, 2019, to January 1, 2020. Patients were divided into two groups. Group A patients were administered a low dose (5 mg per day) of Lisinopril and group B were administered a higher dose of therapy (20 mg/day) for three months. At the end of the study, baseline renal functions, electrolytes, and status of microalbuminuria were compared with follow-up values. The primary outcome was to assess the change in microalbuminuria levels in patients at baseline, one month, and three months of therapy.

Results

A total of 72 patients were included in group A (low dose) and 72 patients were enrolled in group B (high dose). The mean ages of group A and group B were 56.3 ± 12.9 years and 53.48 ± 12.2 years, respectively. The majority of the patients in the groups were male. At baseline, the mean microalbuminuria levels in the two groups were not significantly different however, at three months post treatment, the levels were significantly much lower in high dose patients as compared to patients who were on low dose lisinopril (146.06 ± 23.89 vs. 184.69 ± 26.27; p < 0.0001). The three-month urea levels were significantly lower in group A as compared to group B (38.91 ± 7.07 vs. 43.26 ± 3.02; p = 0.008). Three-month creatinine and potassium levels were not significantly different between the groups (p = 0.7 and 0.12, respectively).

Conclusion

Our study revealed that even though group B (high dose lisinopril) had significantly reduced microalbuminuria, the urea levels were found to be higher in this cohort of patients as compared to group A patients on low-dose lisinopril. Moreover, the majority of the patients in group B reported significant improvements in blood pressure control as compared to group A, which indicated that a high dose of lisinopril is more effective in patients with diabetic nephropathy than a low dose of lisinopril. The levels of creatinine after three months of treatment did not differ significantly. Further randomized trials are warranted in order to ascertain the effectiveness of high dose of lisinopril in patients with diabetic nephropathy.

## Introduction

Diabetes mellitus is a chronic disease that affects all organ systems in our body. There are several macrovascular and microvascular complications associated with diabetes. Some of the micro-complications of diabetes include diabetic nephropathy, neuropathy, and retinopathy [1].

Diabetic nephropathy is the major cause of end-stage renal disease (ESRD) globally, and a prominent cause of diabetes-related morbidity and mortality worldwide [1]. Proteinuria, especially in patients with diabetes, is associated with a markedly reduced survival rate and increased propensity to cardiovascular events [2].

Microalbuminuria refers to a sub-clinical rise in urinary albumin excretion. It corresponds to an albumin excretion rate (AER) of 20-200 µg/min (30-300 µg/d) or an albumin-creatinine ratio (ACR) of 2.5-25 in males and 3.5-35 in females [3]. Since the presence of microalbuminuria is a significant marker for progression to overt nephropathy and adverse cardiovascular events, its reduction is the primary objective for physicians, diabetes experts, and nephrologists alike [4].

It has been observed that there is a very strong pathogenetic link between microalbuminuria and premature death from cardiovascular events. Incipient nephropathy, i.e., the stage of microalbuminuria, usually does not develop within the first five years of diabetes onset; however, it can develop at any time after that, even after 40 years of age [5]. 

A prospective study, the United Kingdom Prospective Diabetes Study (UKPDS), in newly diagnosed type II diabetic patients reported rates of transition from normal to incipient nephropathy of 2% per annum and from incipient to clinical nephropathy of 3% per annum, which are very similar to those seen in patients with type I diabetes [6].

The management of angiotensin-converting enzyme (ACE) inhibitors acts as an additional treatment benefit for patients having type II diabetes and microalbuminuria by reducing overall blood pressure. The findings from three clinical trials of enalapril suggest that the risk of developing clinical proteinuria is significantly reduced among normotensive type II patients with microalbuminuria. In contrast, the study trial of irbesartan showed a reduction of similar risk factors in hypertensive patients only. Moreover, the rate of development of microalbuminuria to clinical proteinuria remained unaffected by intensive vs moderate blood pressure management in another investigation. No conclusive results were obtained when ACE inhibitors were compared with other antihypertensive agents for their antiproteinuric effects and one of the trials revealed that regression was two-fold higher with lisinopril than with nifedipine [7]. Lisinopril is prescribed to manage myocardial infarction, hypertensive disorders, and heart failure as an adjunctive therapy. Previous literature has shown that lisinopril is linked with decreased albumin excretion in urine in patients with essential hypertension [8]. However, there is inconsistency in the existing literature. 

While there are few studies conducted in Pakistan that showed the role of ACE inhibitors in proteinuria, there is none that demonstrate a comparison between low dose and high dose ACE inhibitors, especially with regards to the improvement, i.e., reduction in microalbuminuria levels [9]. Therefore, the present study was conducted to assess the renal effects of high dose versus low dose lisinopril in patients with diabetic nephropathy.

## Materials and methods

A prospective observational study was conducted at the Khyber Teaching Hospital, Peshawar, Khyber Pakhtunkhwa, Pakistan, between July 1, 2019, to January 1, 2020. The ethical approval was obtained from the institutional review board committee of Khyber Teaching Hospital, Peshawar prior to the data acquisition (reference# 5646). 

All patients over the age of 18 years, irrespective of gender, diagnosed with hypertensive disorder, and diabetes mellitus type II were included in the study. Whereas patients who had creatinine levels below 2.5 mg/dL or were on insulin therapy were excluded from the study. A non-probability consecutive sampling technique was used to enroll participants in the study. Patients were divided into two groups. Group A patients were administered a low dose (5 mg per day) of lisinopril, and group B were administered a higher dose of therapy (20 mg/day) for three months. Patients were included in the study after informed consent was obtained from the selected participants. 

The patients were randomly allocated into two groups using a random numbers table. Baseline renal function tests and electrolytes as well as presenting symptoms (blood pressure control, edema, and polyuria) were documented at the start of the study, after one month, and finally at the end of three months. Patients were screened for proteinuria by using conventional dipsticks and then all patients were subjected to 24-hour urinary quantitative analysis using IMMULITE immunoassay (Siemens Healthineers AG, Erlangen, Germany). Then the treatment commenced. All patients were followed at the end of the first month and then at the end of the third month in the outpatient department. Improvements in the microalbuminuria and symptoms were the indicators of the effectiveness of the treatment regime (high dose versus low dose). 

The data analysis was computer-based using IBM SPSS Statistics for Windows, Version 26.0 (Released 2019; Armonk, New York, United States). Descriptive statistics like age and microalbuminuria level were presented as mean and standard deviation while sex, occupation, and side effects were presented as percentage (%) and frequency. The significance of difference between pre- and post-treatment microalbuminuria levels was measured using paired t-tests. Chi-square test was employed to compare side effects between groups with 0.05 as the level of significance.

## Results

A total of 72 patients were included in group A (low dose) and 72 patients were enrolled in group B (high dose). The mean age of group A (low dose) and group B (high dose) were 56.3 ± 12.9 years and 53.48 ± 12.2 years, respectively. The majority of the patients in the groups were male. Majority of the patients in both groups were presented in the outpatient department (OPD) (Table 1). Only about 23 (31.9%) of patients were admitted in the department for management. 

**Table 1:** Baseline characteristics of study participants

Parameters	Group A (Low Dose)	Group B (High Dose)
Age (years)	56.3 ± 12.9	53.48 ± 12.2
35-45	16 (22.2%)	8 (11.1%)
46-55	19 (26.4%)	31 (43.1%)
56-65	23 (31.9%)	21 (29.2%)
> 65	14 (19.4%)	12 (16.67%)
Mode of patient presentation		
Outpatient department	49 (68.1%)	35 (48.6%)
Inpatient department	23 (31.9%)	37 (51.4%)
Gender		
Male	53 (73.6%)	41 (56.9%)
Female	19 (26.4%)	31 (43.1%)

Table 2 illustrates the comparative biochemical variables between group A (low dose) and group B (high dose) at baseline and three months post-treatment. At baseline, the mean microalbuminuria levels in the two groups were not significantly different; however, at three months post treatment, the levels were significantly much lower in high-dose patients (< 0.0001). The three-month urea levels were significantly lower in group A (low dose) as compared to group B (high dose) (p = 0.008). Three-month creatinine and potassium levels were not significantly different between the groups (p = 0.7 and 0.12, respectively). 

**Table 2:** Pre- and post-treatment laboratory parameters

Parameter	Group A (Low Dose)	Group B (High Dose)	p-value
Microalbuminuria			
Baseline	236.34 ± 28.39	228.49 ± 28.61	0.107
Three-months	184.69 ± 26.27	146.06 ± 23.89	< 0.0001
Urea level			
Baseline	35.57 ± 4.53	34.77 ± 4.5	0.41
Three-months	38.91 ± 7.07	43.26 ± 3.02	0.008
Creatinine level			
Baseline	0.95 ± 0.22	0.93 ± 0.22	0.38
Three-months	1.01 ± 0.26	1.06 ± 0.3	0.7
Potassium level			
Baseline	3.91 ± 0.47	3.81 ± 0.38	0.9
Three-months	4.15 ± 0.45	4.45 ± 0.41	0.12

Over 90% of patients in group B reported improvements in the symptoms (blood pressure control, edema, and polyuria) thus indicating that treatment was significantly more effective in high dose of lisinopril as compared to low dose of lisinopril (p = 0.002) (Table 3).

**Table 3:** Comparison of efficacy between low-dose and high-dose lisinopril groups

Effectiveness	Group A (low dose)	Group B (high dose)	p-value
Yes	43 (59.9%)	66 (90.6%)	0.002
No	29 (40.1%)	6 (8.4%)	

Table 4 illustrates the effect of low dose lisinopril as compared to high dose lisinopril. There were significant reductions in both groups. High doses of lisinopril were more effective in decreasing systolic and diastolic blood pressure. 

**Table 4:** Effect of low-dose versus high-dose lisinopril on blood pressure

Group	Baseline	Three month Follow-up	P-value
Systolic Blood Pressure			
Group A (Low Dose)	156.93 ± 7.62	153.18 ± 7.09	0.002
Group B (High Dose)	156.7 ± 9.12	142.5 ± 9.46	0.0001
Diastolic Blood Pressure			
Group A (Low Dose)	105.59 ± 6.48	102.15 ± 8.47	0.007
Group B (High Dose)	103.9 ± 6.54	79.59 ± 2.83	0.0001

## Discussion

The generic formulation of lisinopril is known to reduce microalbuminuria [9]. A dose-based comparison was uncommon to find. McConnell and colleagues performed a study to assess the clinical pharmacy specialist implementation of lisinopril therapy in patients with coronary artery disease and diabetes mellitus [10]. The study was conducted among patients suffering from both diseases in coordination with pharmacy services. The trial intended to increase the proportion of patients receiving the dose of 20 mg/day or the maximum acceptable therapeutic dose of the angiotensin-converting enzyme (ACE) inhibitor, lisinopril. We found that higher doses were significantly more effective in reducing microalbuminuria.

The study by McConnell et al. [10] had a few differences from our study, especially with regard to the number of patients and the main outcome measures. Our main outcome measure was a reduction in microalbuminuria levels, which was not highlighted in this study. The generic used, the dosage plan (a low-dose or control group vs. a treatment or high-dose group), and the format of follow-up and lab review were comparable. Current evidence supports our findings whereby higher doses of ACE inhibitors are superior in reducing the risk of cardiovascular events [11,12].

In another review, the relationship between lisinopril and cardiovascular outcomes was assessed [12]. The review revealed that high doses of lisinopril significantly reduced the risk of overall morbidity and mortality as compared to a low dose of lisinopril [12]. Majumdar et al. investigated the added benefits of higher doses of digoxin, β-blockers, and angiotensin-converting enzyme inhibitors (ACEIs) in comparison to standard dosages. Additionally, the advantages of higher dosages with regard to these medications were also studied [13]. The research was a secondary data analysis of an active-comparator, randomized, controlled experiment. A study was carried out in 287 facilities across approximately 20 countries and was based on the comparison between two doses of lisinopril (33.2mg/day and 4.5mg/day) [14]. It was revealed that the composite end-point declined iteratively upon continuous administration of ACE inhibitors in higher dosages alone (p = non-significant, adjusted odds ratio 0.93, n = 475), with β-blockers (p = non-significant, adjusted odds ratio 0.89, n = 72), and with β-blockers and digoxin (p = 0.006, adjusted odds ratio 0.47, n = 77) in contrast to ACE inhibitors in lower dosages [14].

Concisely, current evidence demonstrates the benefits of using combinations of high-dose ACE inhibitors, β-blockers, and diuretics in significantly reducing death and disability when compared to conventional therapy for patients with congestive heart failure [14]. As current guidelines suggest that microalbuminuria is an independent indicator of stroke and other cardiovascular events, it is absolutely necessary to explore optimum intervention strategies to reduce microalbuminuria in patients with diabetes mellitus [15]. Elsafa et al. studied the impact of ace inhibitors on reducing the occurrence of microalbuminuria in patients with diabetes [15]. The study concluded that patients who cannot tolerate ACE inhibitors should be switched to angiotensin receptor blockers (ARBs). 

Another study recently conducted was a randomized and double-blinded clinical trial in which combined therapy of lisinopril and valsartan was compared with high-dose monotherapies in patients with microalbuminuria [16]. Another trial concluded that despite the fact that both treatments were effective in reducing the rate of microalbuminuria, the combined effect of valsartan and lisinopril superseded the high-dose administration of lisinopril [11]. 

Similarly, some other studies also explored the use of lisinopril in patients presenting with microalbuminuria and reached the same conclusion [17]. In nutshell, a higher dose of lisinopril renders significant benefits to patients with both microalbuminuria with a higher reduction in microalbuminuria patients. Higher than average doses do better than low doses without any marked untoward effects.

There are certain limitations in the study. For instance, due to a limited sample size, the study findings cannot be generalized to a larger population. Therefore, further large-scale and multicentered studies are needed to evaluate the optimum dose for the highest efficacy in these patients. 

## Conclusions

Our study revealed that even though group B (high dose lisinopril) had significantly reduced microalbuminuria, the urea levels were found to be higher in this cohort of patients as compared to the group A patients on low dose lisinopril. Moreover, the majority of the patients in group B reported significant improvements in blood pressure control as compared to group A, which indicated that a high dose of lisinopril is more effective in patients with diabetic nephropathy than a low dose of lisinopril. The levels of creatinine after three months of treatment did not differ significantly. Further randomized trials are warranted in order to ascertain the effectiveness of a high dose of lisinopril in patients with diabetic nephropathy.
